# Loss of *flrt2* gene leads to microphthalmia in zebrafish

**DOI:** 10.1242/bio.059784

**Published:** 2023-06-15

**Authors:** Siyu Yang, Lianggui Huang, Huiling Liang, Jingyi Guo, Liyue Liu, Shuyi Chen, Mingzhe Cao

**Affiliations:** ^1^ Department of Ophthalmology, The Seventh Affiliated Hospital of Sun Yat-Sen University, Shenzhen, 518107, China; ^2^ State Key Laboratory of Ophthalmology, Zhongshan Ophthalmic Center, Sun Yat-sen University, Guangzhou, 510060, China; ^3^ China Zebrafish Resource Center, National Aquatic Biological Resource Center, Institute of Hydrobiology, Chinese Academy of Sciences, Wuhan, 430072, China

**Keywords:** *flrt2*, Retinal, Zebrafish, Microphthalmia, CRISPR/Cas9

## Abstract

As a member of the *fibronectin leucine-rich transmembrane* (*flrt*) gene family, *fibronectin leucine-rich transmembrane 2* (*flrt2*) is strongly expressed in a subset of sclerotome cells, and the resultant protein interacts with FGFR1 in the FGF signaling pathway during development. Studies on *flrt2* have focused mainly on its roles in the brain, heart and chondrogenesis. However, reports on its expression and function in the zebrafish retina are lacking. Here, we detected the high expression of *flrt2* in zebrafish retina using *in situ* hybridization technique and developed an *flrt2*-knockout (KO) zebrafish line using CRISPR/Cas9 genome editing. Quantitative real-time PCR was used to measure the expression levels of *flrt2*, which results in an approximately 60% mRNA reduction. The *flrt2*-KO zebrafish eyes’ altered morphological, cellular, and molecular events were identified using BrdU labeling, TUNEL assay, immunofluorescent staining, fluorescent dye injection and RNA sequencing. Abnormal eye development, known as microphthalmia, was found in *flrt2*-KO larvae, and the retinal progenitor cells exhibited increased apoptosis, perhaps owing to the combined effects of *crx*, *neurod4*, *atoh7*, and *pcdh8* downregulation and *Casp3a* and *Caspbl* upregulation. In contrast, the retinal neural development, as well as retinal progenitor cell differentiation and proliferation, were not affected by the *flrt2* deletion. Thus, *flrt2* appears to play important roles in retinal development and function, which may provide the basis for further investigations into the molecular mechanisms of retinal development and evolution.

## Teaser

**Summary:** *flrt2* is necessary for the normal development of the zebrafish eyes since it is strongly expressed in the retina and deletion of *flrt2* causes microphthalmia in zebrafish

## INTRODUCTION

Congenital malformations of the eyes are one of the leading causes of childhood blindness. The most serious of these malformations are anophthalmia, microphthalmia, and coloboma, which account for up to 11% of the cases of blindness in children (Williamson and FitzPatrick, 2014). Among them, microphthalmia refers to the reduction of eyeball volume, and it may be associated with coloboma or an orbital cyst. The main manifestation of ocular coloboma is the absence of ventral tissue (mainly retina). These conditions typically result from a genetic abnormality, maternal illness, teratogenic exposure, or vitamin deficiency. Advances in our understanding of the molecular mechanisms governing eye development, combined with improved genetic diagnosis techniques, have resulted in the identification of many genes that, when disrupted, result in malformations (Plaisancié et al., 2019). Despite these advances, our understanding of eye disease progression remains patchy, and most of the molecular causes underlying eye malformations remain unknown. Animal model research is critical to understanding retinal development under both normal and pathological conditions (Kimmel et al., 1995). Zebrafish is an effective animal model to research human retina due to their rapid genetic modification, simple real-time observability, greater fecundity compared with mice, and similar retinal structure to that of humans (Ota and Kawahara, 2014; Richardson et al., 2017; Varshney et al., 2015b). Gene-editing technologies such as the ZFNs (Doyon et al., 2008), TALENs (Huang et al., 2011), and CRISPR/Cas9 (Jao et al., 2013; Liu et al., 2014) have been developed in recent years to knock out genes in zebrafish (Varshney et al., 2015a). The CRISPR/Cas systems are especially appealing for zebrafish gene function screening because they may be used to easily introduce mutations at nearly any loci, which increases the ability to investigate the etiology and pathological progression in disease models (Moreno-Mateos et al., 2015; Xu and Qi, 2019).

**Fig. 1.**
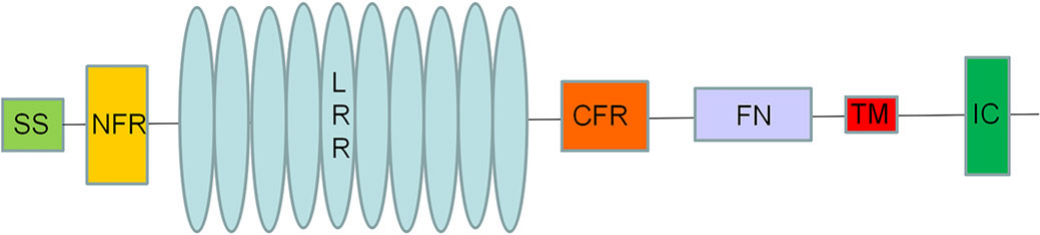
**Schematic diagram illustrating the primary structure of FLRT2 adapted from** **Flintoff et al., 2014** **and** **Lacy et al., 1999****.** The extracellular domain contains 10 Leucine Rich Repeat sequences flanked by N- and C- terminal cysteine-rich regions. The FNIII domain lies closer to the cell membrane. These proteins span the cell membrane once and have a short cytoplasmic tail. SS: signal sequence; NFR: cysteine-rich domains flanking N′ terminal ends of LRR domain; LRR: 10 leucine-rich repeats; CFR: cysteine-rich domains flanking C′ terminal ends of LRR domain; FN: fibronectin III domain; TM: transmembrane domain; IC: cytoplasmic tail.

*flrt2* belongs to the fibronectin leucine-rich transmembrane family, which encode putative type I transmembrane proteins. Each of which contains 10 leucine-rich repeats (LRRs) flanked by N-terminal and C-terminal cysteine-rich regions, type III fibronectin-domain (FNIII), and transmembrane domains with a short intracellular tail (Lacy et al., 1999) (Fig. 1). To date, the evidence suggests different roles for the domains found in the FLRT proteins, as follows: the LRR domain plays a role in homotypic cellular recognition; the FNIII domain is involved with binding to receptors of *Fgf*; and the intracellular domain is responsible for modulating *Fgf* receptor signaling in both mice and *Xenopus* (Haines et al., 2006; Karaulanov et al., 2006). Proteins containing these domains are involved in many biological processes, including embryonic development, axonal formation, retinal cells development and neuronal survival. FLRT proteins are also referred to as cell adhesion molecules (CAMs), and they interact with heterophilic receptors, acting as guiding factors in the early stages of embryonic, neuronal, and vascular development (Xu et al., 2011). FLRT2 has been found in the pancreas, heart, skeletal muscle, and brain, and it plays crucial roles in excitatory neuron development, such as neuron migration, axon guidance, and synapse formation. It also acts as a chemorepellent in axon guidance and cell migration through interactions with uncoordinated-5 (Unc5) receptors, as well as functioning in cell adhesion through interactions with latrophilins (Flintoff et al., 2014). During mouse eye development, *Flrt2* is expressed in the optic cup of E10.5 embryos and is expressed more at the edges of the neural layer. Additionally, at E15.5, *Flrt2* was found to express in the epithelial lining of the eye lid (prior to fusion of upper and lower eyelids) and the equatorial region of the lens (Gong et al., 2009). *Flrt2* was determined to be very selectively expressed in retinal precursor cells in these earlier investigations (Visser et al., 2015), which raises the possibility that it plays a significant regulatory role in the growth and physiological operation of the retina. However, to our knowledge, the *flrt2* gene has never been linked to coloboma or microphthalmia.

In this report, we created and described a novel zebrafish *flrt2* mutant line to further characterize the effects of *flrt2* on the development of, and physiological function in, zebrafish retina. We determined that *flrt2* is crucial for the growth and function of the retina. Further investigations explored the regulatory mechanism that may incorporate *flrt2*. This study will help increase our understandings of the mechanisms that control retinal growth and its physiological functions, as well as providing a theoretical foundation for the detection, prognostic evaluation, and treatment of ocular defects.

## RESULTS

### *flrt2* expression in the developing retina of zebrafish

**Fig. 2.**
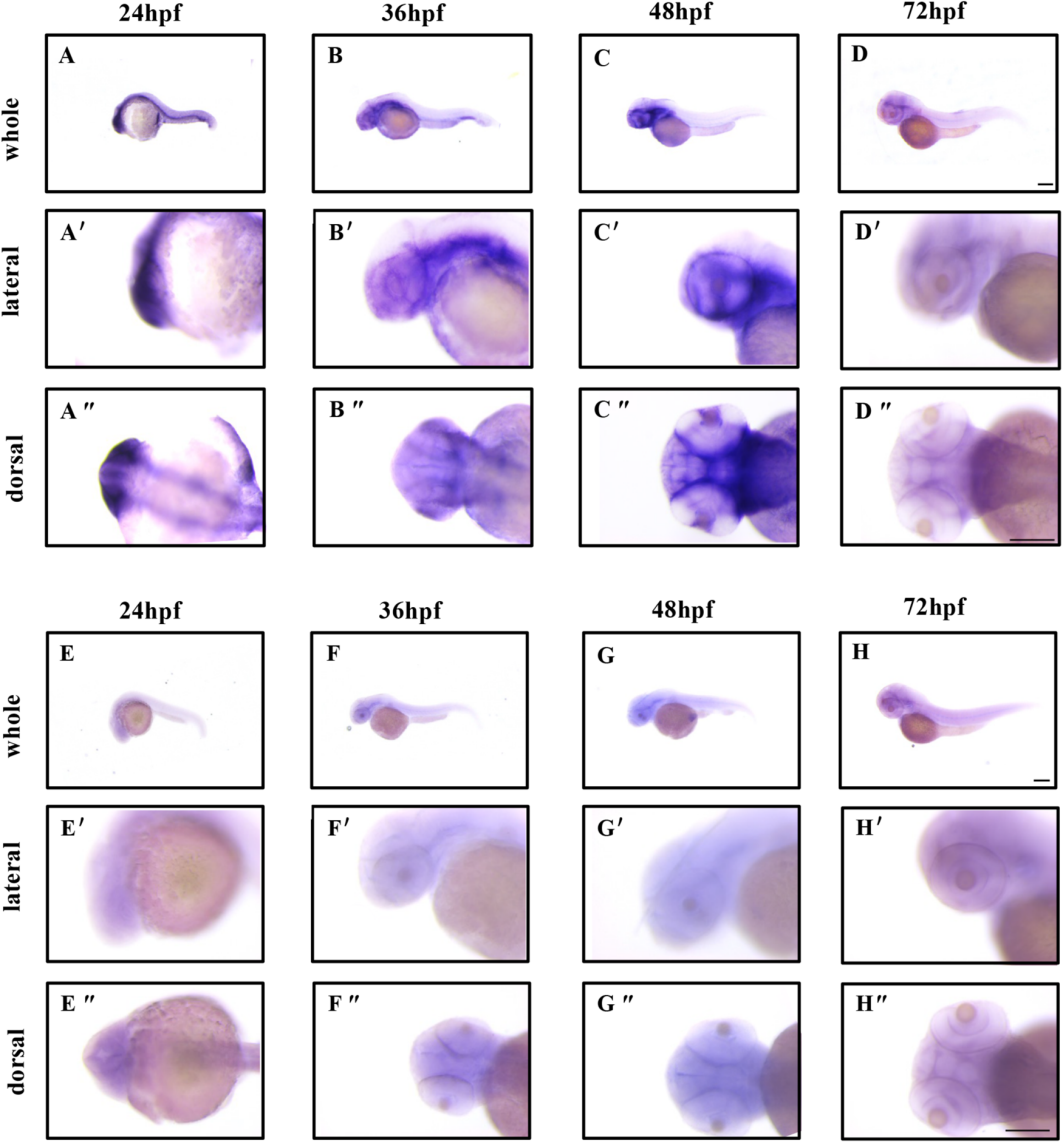
***flrt2* is expressed in developing zebrafish eyes.** (A,D″) Lateral views of *flrt2* mRNA whole-mount *in situ* hybridization of (A,A′) 24 hpf, (B,B′) 36 hpf, (C,C′) 48 hpf, and (D,D′) 72 hpf zebrafish embryos. (A″-D″) Dorsal views of *flrt2* mRNA whole-mount *in situ* hybridization of (A″) 24 hpf, (B″) 36 hpf, (C″) 48 hpf, and (D″) 72 hpf zebrafish embryos. (E-H″) Embryos exposed to a sense riboprobe were used as a negative control. *n*=20/each group. Scale bar: 200 µm. Leica stereomicroscope M205 was used: objective: M205FA 30×/161×; scaling (per pixel): 1.00 pixel×1.00 pixel; image size (pixels): 1392×1040.

Prior to examining the roles played by *flrt2* throughout zebrafish retinal development, we first used whole-mount *in situ* hybridization (WISH) to characterize *flrt2* expression patterns in zebrafish embryonic eyes during the primary phase of zebrafish retinal development. And the results showed that at 24 h post fertilization (hpf), *flrt2* expression was detected in neurons, lens and notochord (Fig. 2A-A″). Then, for 36 to 48 hpf expression continued in the neurons of the telencephalon as well as cells dorsal to the lens (Fig. 2B-C″). However, at 72 hpf, *flrt2* expression was minimal in the brain and eye (Fig. 2D-D″). The WISH results using negative controls are shown in Fig. 2E-H″. The description of the expression pattern is only partially complete owing to the probe's inability to access some structures, such as the notochord, the majority of the trunk, and the tail. Based on the expression pattern of *flrt2*, we decided to investigate its effects on neurodevelopment and eye development.

### Generation and characterization of a *flrt2*-knockout (KO) zebrafish line

**Fig. 3.**
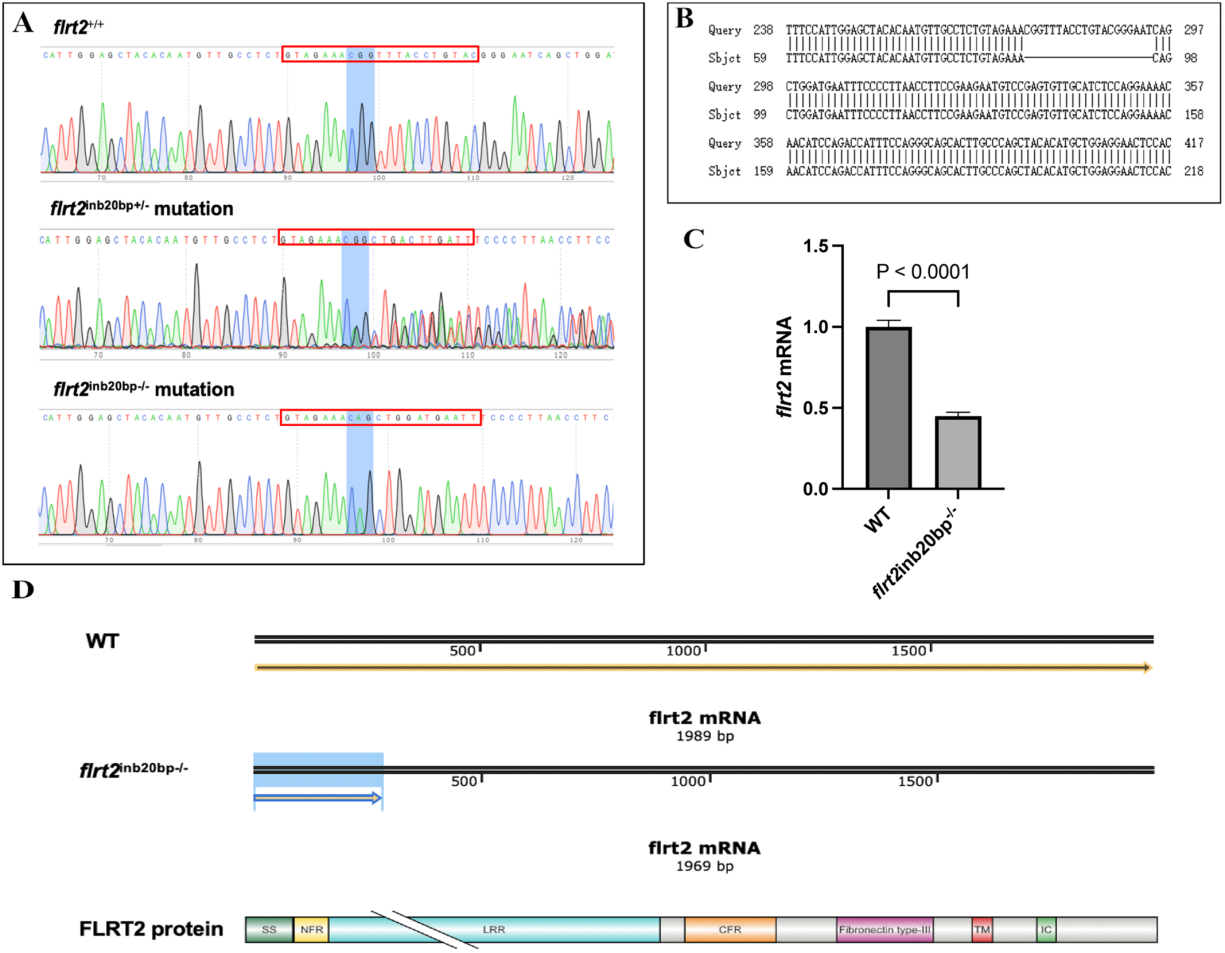
**Generation of *flrt2*-KO zebrafish line by CRISPR/Cas9 genome editing.** (A) Sequencing maps of WT, *flrt2*^+/−^ mutants and *flrt2*^−/−^ mutants. The sequences in the red frame represent the areas targeted by CRISPR/Cas9. Blue background indicates CGG base pairs in WT and heterozygous zebrafish. (B) Sequence alignment of the WT and *flrt2*-KO zebrafish line, including the 20-bp deletion in homozygotes. (C) Reduced expression of *flrt2* mRNA in the brain of WT and *flrt2*^−/−^ larvae (24 hpf) zebrafish analyzed by RT-qPCR (*n*=30/each group); *t*=23.58, *P*<0.0001. The solid bars represent the means±standard deviations. (D) Schematic diagram of FLRT2 protein translation. The yellow arrows indicate the process and direction of *flrt2* mRNA translation into protein. Blue background means the position of the premature termination of protein translation. And the bottom indicates that the FLRT2 protein translation is forced to prematurely terminate the site.

We generated mutants by co-injecting Cas9 mRNA and gRNA into zebrafish embryos (one-cell stage) to evaluate the function of *flrt2* during the development of the zebrafish retina. The *flrt2*-targeted allele carried a deletion of 20 bp as determined by DNA sequencing of target-specific PCR products (Fig. 3A,B). Two methods were employed to assess the stability of the *flrt2^−/−^* mutant line. First, we used RT-qPCR to measure the mRNA levels in inbred F3 homozygotes’ offspring (RT-qPCR using mRNA derived from larval brains at 24 hpf). The RT-qPCR revealed that *flrt2* expression was approximately 60% lower than wild-type (WT) levels (Fig. 3C). Through analysis, FLRT2 protein translation was found to terminate prematurely after the deletion of 20 bp (Fig. 3D). Then, we used western blotting to assess FLRT2 protein expression. Unfortunately, three antibodies (anti-FLRT2: ab154023, AF2877S, and ABP58572) that were used to investigate the level of FLRT2 protein expression, but they did not recognize the FLRT2 protein. Thus, the successful generation of a mutant *flrt2*-deficient zebrafish line was based on the RT-qPCR results.

### *flrt2-*KO leads to microphthalmia in zebrafish

**Fig. 4.**
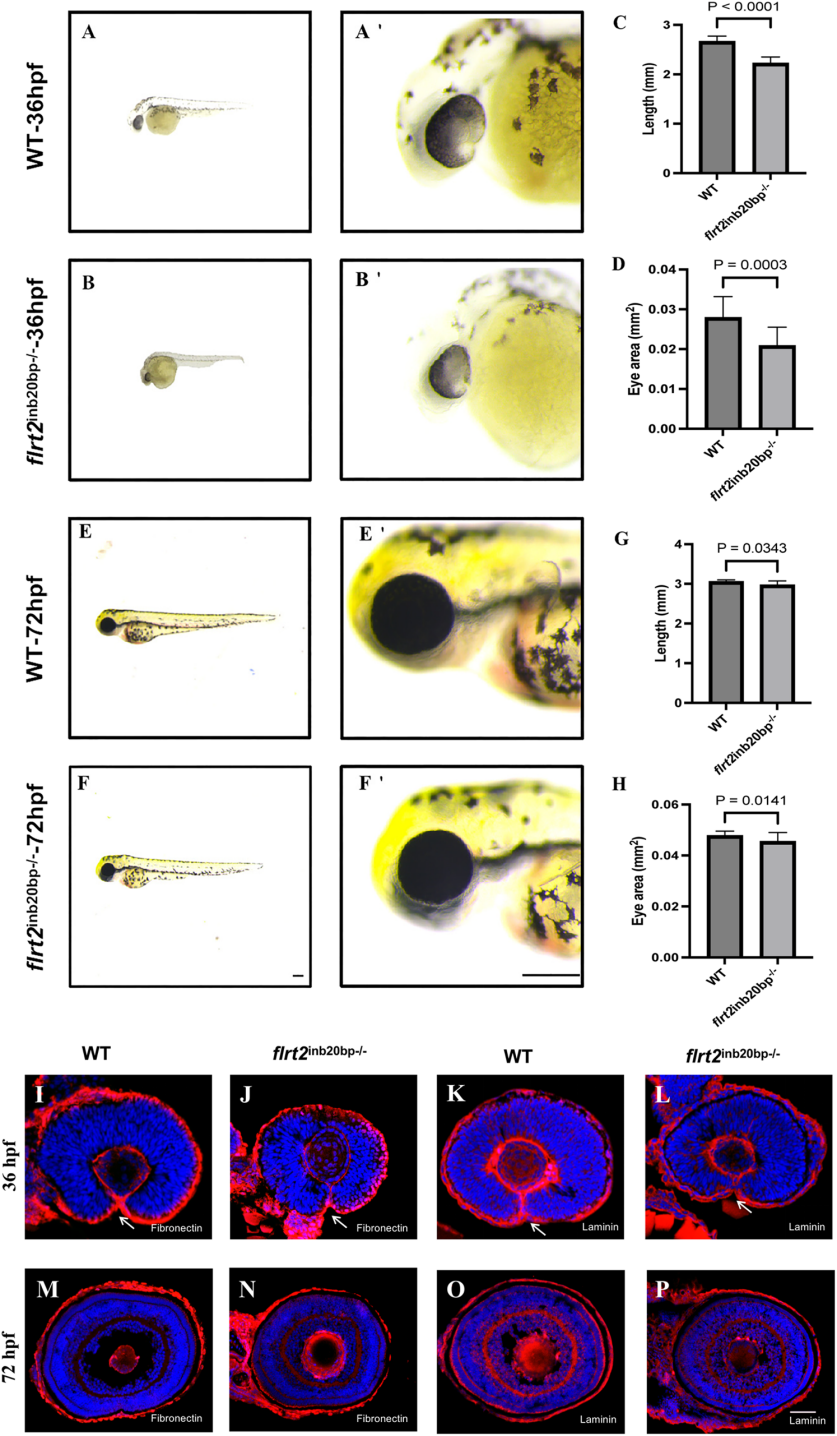
***flrt2*-KO leads to zebrafish slow development and reduces eye size.** (A,B) Phenotypes of WT and *flrt2*^−/−^ embryos at 36 hpf, with A′ and B′ are the magnified images of the heads from A and B, respectively. (C,D) Statistical analysis of body length and eye size in WT and *flrt2*^−/−^ embryos at 36 hpf (*n*=17/each group); C: *t*=10.543, *P*<0.0001; D: *t*=4.098, *P*=0.0003. (E,F) Phenotypes of WT and *flrt2*^−/−^ embryos at 72 hpf, with E′ and F′ showing the magnified images of the heads from E and F, respectively. Leica stereomicroscope M205 was used: M205FA 30×/161×; scaling (per pixel): 1.00 pixel×1.00 pixel; image size (pixel): 1392×1040. Scale bars: 200 µm. (G,H) Statistical analysis of body length and eye size in WT and *flrt2*^−/−^ embryos at 72 hpf (*n*=20/each group); G: *t*=2.290, *P*=0.0343; H: *t*=2.575, *P*=0.0141. The solid bars represent the means±standard deviations. (I–P) At 36 hpf and 72 hpf, WT zebrafish were stained with (I,M) anti-fibronectin or (K,O) anti-laminin. At 36 hpf and 72 hpf, *flrt2*^−/−^ zebrafish were stained with (G,N) anti-fibronectin or (L,P) anti-laminin (*n*=20/each group). Arrows point to the unfused OF. LSM 980 with Airyscan was used: objective: plan-apochromat 20×/0.8 M27; scaling (per pixel): 0.414 µm×0.414 µm; image size (pixel): 1024×1024; effective NA: 0.8. Scale bar: 50 µm.

To better understand the function of *flrt2* in zebrafish eye development, we assessed eye phenotypes and measured eye sizes and body lengths at 36 hpf in *flrt2*-KO and WT larvae. At 36 hpf, *flrt2*^−/−^ zebrafish showed significantly smaller eyes and a shorter body length when compared with WT zebrafish (Fig. 4A-D). However, over time, these variations in the broad phenotypes gradually disappeared (Fig. 4E-H). Next, we determined whether there were other phenotypes besides microphthalmia, such as coloboma. Thus, we marked the basement membrane with anti-laminin and anti-fibronectin immunostaining to determine whether coloboma appeared in the zebrafish eyes. At 36 hpf, laminin^+^/fibronectin^+^ basement membranes had developed from the ventral optic cup (OC) in the eyes of WT and *flrt2*^−/−^, and the indentation indicating the location of the optic fissure (OF) had also developed (Fig. 4I-L, arrows). At 72 hpf, the OF had already fused to form a complete OC, the laminin^+^/fibronectin^+^ basement membrane remained intact in WT and *flrt2*^−/−^ larvae (Fig. 4M-P).

### Effects of *flrt2*-KO on retinal cell proliferation and survival

**Fig. 5.**
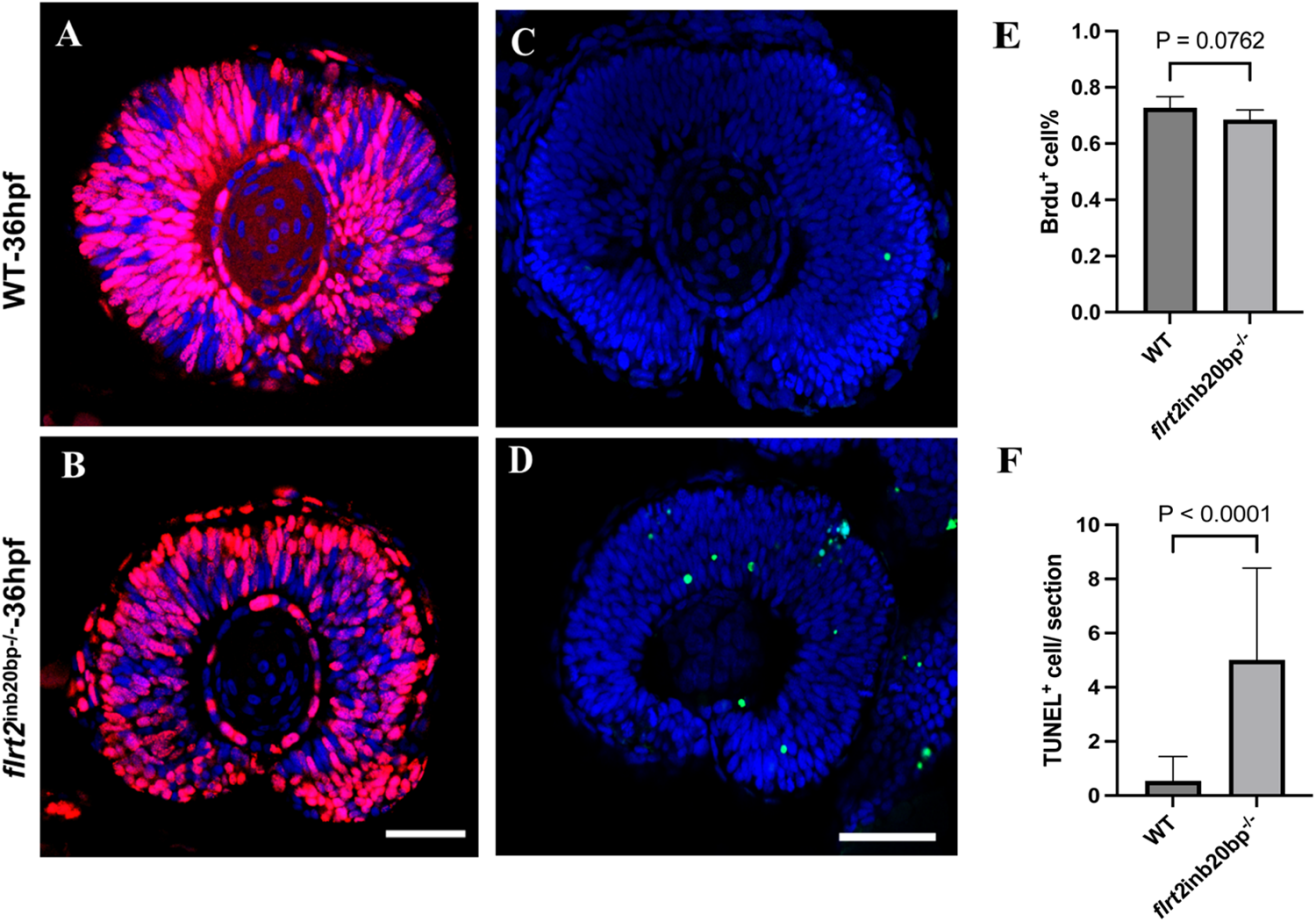
**Retinal progenitor cell proliferation was not affected by the *flrt***-**KO.** (A,B) Images of 36 hpf zebrafish eye sagittal sections stained with anti-BrdU antibody (red). (C) Quantification of BrdU^+^ cells/section in WT and *flrt2*-KO retinas at 36 hpf; *t*=1.977, *P*=0.0762. Scale bars: 50 µm (*n*=6/each group). (D,E) TUNEL staining (green) of 36 hpf zebrafish eye sagittal sections. (F) Quantification of TUNEL^+^ cells/section in WT and *flrt2*-KO retinas at 36 hpf; *t*=4.911, *P*<0.0001. Scale bars: 50 µm (*n*=15/each group). The solid bars represent the means±standard deviations. LSM 980 with Airyscan was used: objective: plan-apochromat 20×/0.8 M27; scaling (per pixel): 0.414 µm×0.414 µm; image size (pixel): 1024×1024; effective NA: 0.8.

Because proper regulation of cell proliferation is essential for retinal development, we investigated whether cell proliferation was affected in *flrt2*-KO zebrafish in the retinas at 36 hpf. Live 34 hpf zebrafish embryos were bromodeoxyuridine (BrdU)-labeled for 2 h, and the proportion of BrdU^+^ cells in the middle of each eye was measured. Approximately 72% of retinal progenitor cells were BrdU^+^ in the control eyes, whereas the percentage of BrdU^+^ cells in *flrt2*^−/−^ mutant retinas was less, at approximately 68% (Fig. 5A,B), but there was no significant difference between the two groups (Fig. 5C). These findings indicating that cell proliferation was not affected by the *flrt2*-KO.

Next, we identified apoptotic cells using the TUNEL assay. We chose one middle sagittal segment representative of each eye for cell counting to ascertain whether the *flrt2*-deficiency influenced cell apoptosis in the retinas. As shown in Fig. 5D-F, the retinal progenitor cell (RPC) population in WT retinas at 36 hpf contained a small number of apoptotic cells; only one or two apoptotic cells per segment. However, massive apoptosis was observed in the retinas of the *flrt2*-KO group, with a significant difference in the number of apoptotic cells compared with the WT group (*P*<0.05). These findings showed that cell survival in the retinas was substantially hampered, which may account for the microphthalmia phenotype in *flrt2*-KO zebrafish.

### Effects of the *flrt2*-KO on retinal neuronal differentiation

**Fig. 6.**
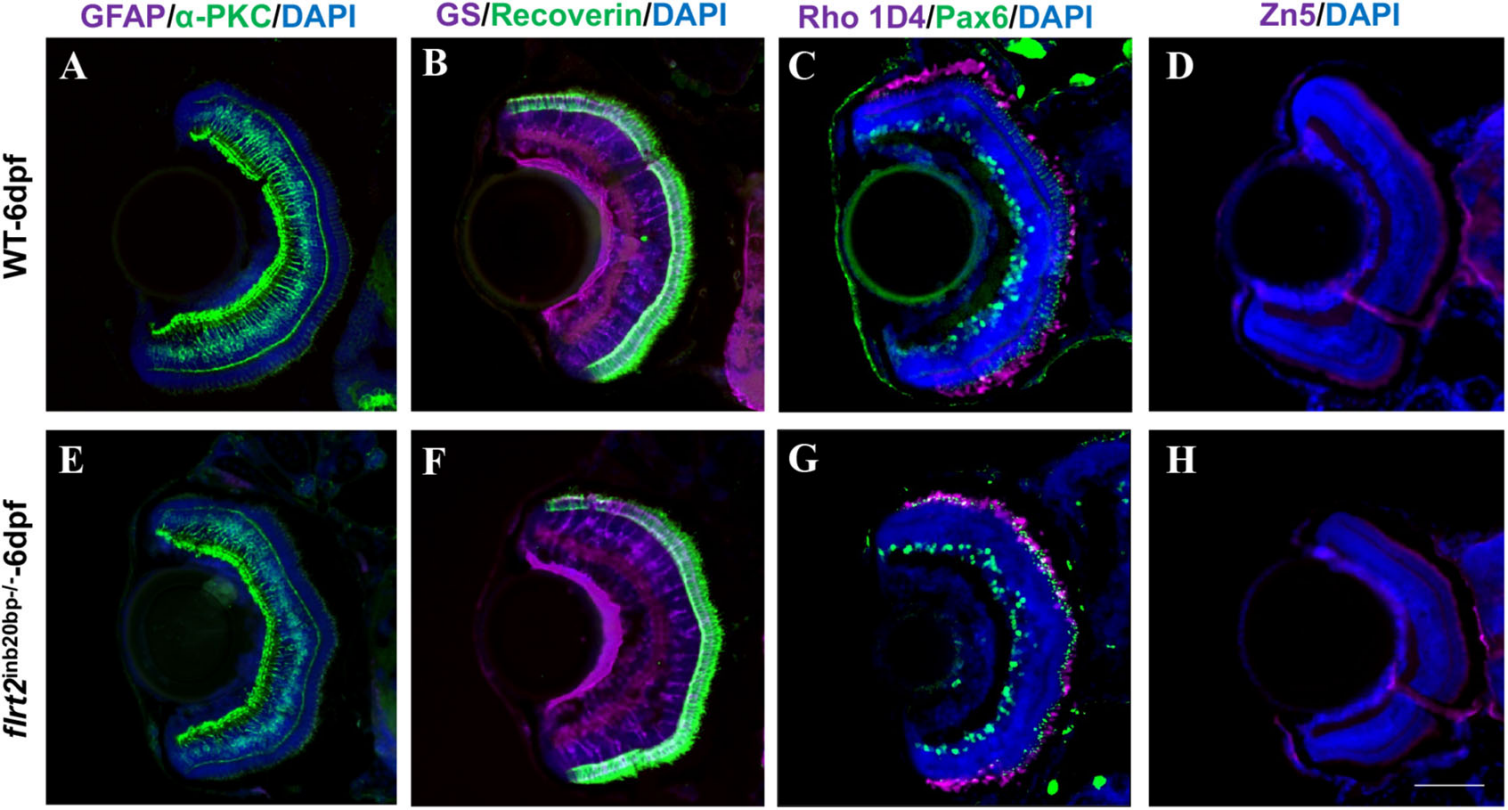
**Retinal cells fate determination in *flrt2***-**KO zebrafish.** Immunofluorescence staining with retinal neural markers: (A,E) co-labelling with anti-GFAP antibodies (for glial cells, magenta) and anti-α-PKC antibodies (for bipolar cells, green). (B,F) Co-labelling with anti-GS antibodies (for Müller cells, magenta) and anti-Recoverin antibodies (for photoreceptor cells, green). (C,G) Co-labelling with anti-Rho 1D4 antibodies (for long double-cone outer segments, magenta) and anti-Pax6 antibodies (for ganglion and amacrine precursor cells, green). (D,H) Labelling with anti-Zn5 antibodies (for mature RGCs, magenta) in WT and *flrt2*-KO larvae retinas. Blue, DAPI staining of the nuclei. *n*=20/each group. Scale bar: 50 μm. ISM 710 was used: objective: LD plan-neofluar 20×/0.4 Korr M27; scaling (per pixel): 0.497 µm×0.497 µm; image size (pixel): 1388×1040; effective NA: 0.4; depth of focus: 8.02 µm.

Since microphthalmia is a common phenotype associated with retinal degeneration in zebrafish, we used immunofluorescence labeling to investigate whether *flrt2*-deficient embryos showed abnormal retinal cell development. Sections of WT and *flrt2*^−/−^ mutant larvae were immunolabeled at 6 dpf with a variety of antibodies, including an anti-Zn5 antibody for mature RGCs, an anti-Pax6 antibody for ganglion cells and amacrine precursor cells, an anti-α PKC antibody for bipolar cells, an anti-GS antibody for Müller cells, an anti-GFAP antibody for glial cell, an anti-Recoverin antibody for photoreceptor cells, and an anti-Rho 1D4 antibody for long double-cone outer segments, to determine the impacted principal retinal cell classes. The findings demonstrated that *flrt2*-KO zebrafish contained all retinal cell types (Fig. 6). In addition, there were no differences between *flrt2*^−/−^ mutants and WT larvae, indicating that the *flrt2* mutation had no effect on how any retinal cell type matured.

### Axon projection in *flrt2*-KO zebrafish

**Fig. 7.**
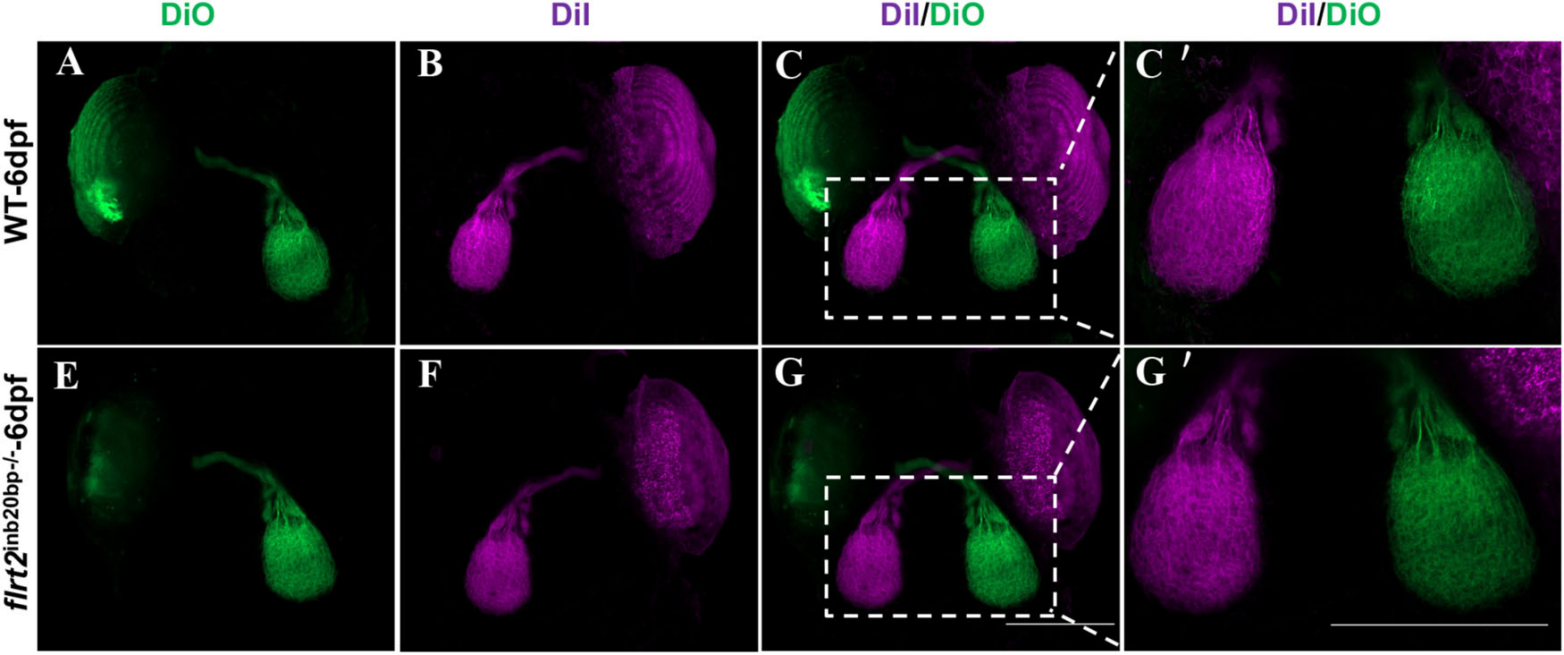
***flrt2*-KO zebrafish showed normal optic nerve projection.** RGC axon projection in zebrafish at 6 dpf. Above: RGC axon projection in WT larvae, below: RGC axon projection in *flrt2*^−/−^ mutant larvae. Retinotopic anterograde RGC axon labeling using DiI and DiO. *n*=20/each group. Scale bar: 200 μm. (A-G) LSM 980 with Airyscan was used: objective: plan-apochromat 10×/0.45 M27; scaling (per pixel): 0.829 µm×0.829 µm×8.790 µm; image size (pixel): 1024×1024; effective NA: 0.45; depth of focus: 5.43 µm. (C′,G′) LSM 980 with Airyscan was used: objective: plan-apochromat 20×/0.8 M27; scaling (per pixel): 0.414 µm×0.414 µm×3.760 µm; image size (pixel): 1024×1024; effective NA: 0.8; depth of focus: 1.72 µm.

The RGC axons that project to the tectum in *flrt2*-KO zebrafish at 6 dpf were then traced to further examine whether *flrt2* is involved in RGC axon projection in zebrafish. The larvae were immobilized, and then dye (DiI and DiO) was unilaterally injected to label one side of the projected axons, such that the merged images would clearly show axonal decussation. We discovered that both the eyes of *flrt2*-KO zebrafish and those of the control zebrafish displayed RGC axons from the retina that projected exclusively to the contralateral tectum. They also displayed symmetric and intact axonal decussation, RGC axon-filled tecta, and typical fascicle-branched optic tracts (Fig. 7), indicating that the RGC axon projections were normal.

### Gene expression changed in *flrt2*-KO zebrafish eyes

**Fig. 8.**
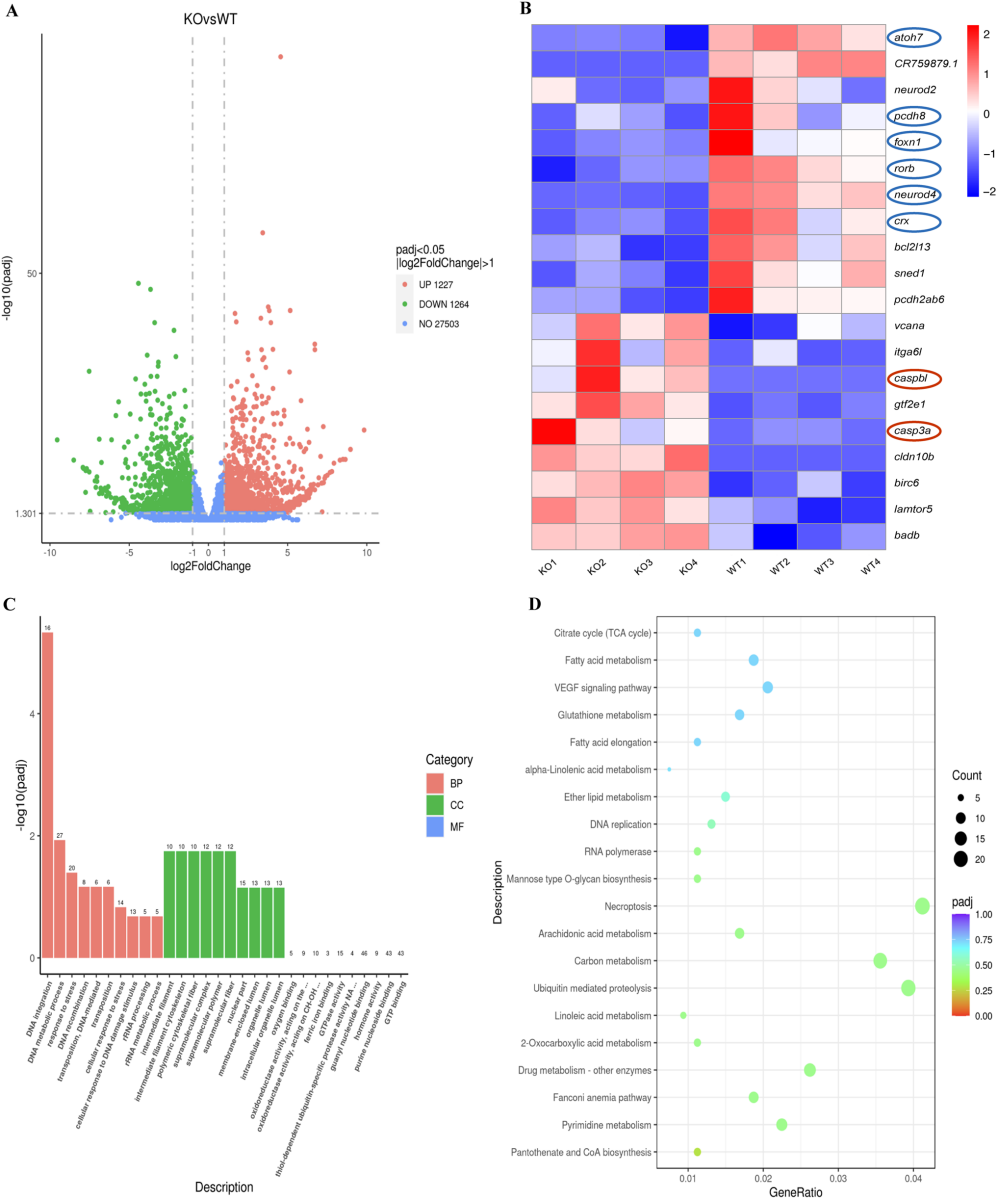
**Gene expression changes in *flrt2*-KO eyes.** (A) Volcano plots showing the gene expression differences between *flrt2*-KO and WT retinas at 36 hpf identified ueing RNA-seq. (B) RNA-seq heatmap showing the DEGs associated with cell adhesion, cell apoptosis, retinal development, and transcriptional regulatory activity in the *flrt2*-KO and WT eyes (*n*=40 per group). The heatmap shows upregulated and downregulated genes in red and blue, respectively. The color key represents the normalized data from RNA-seq. (C) Gene ontology enrichment analysis like biological process (BP), cellular component (CC) and molecular functions (MF). (D) Functional pathway enrichment analysis. The DEGs were involved in various KEGG biological pathways.

RNA sequencing (RNA-seq) and data analyses of the transcriptome profiles in *flrt2*-KO and control zebrafish eyes were carried out to investigate whether the *flrt2*-KO altered gene expression in developing zebrafish eyes (RNA derived from retinas of larvae at 36 dpf, eight samples, *n*=40 per sample). A total of 2492 genes, including 1227 upregulated and 1264 downregulated genes, had altered expression levels, according to differential gene expression (DEG) analyses (Fig. 8A). A critical element in apoptosis is the activation of the caspases family. We discovered that the apoptotic cascade pathway's essential components, *Casp3a* and *Caspbl*, were markedly upregulated in the *flrt2*-KO zebrafish eyes. Additionally, the enhanced apoptotic activity shown in mutant eyes may be brought on by this upregulation. *pcdh8* and several retinal neurogenesis regulatory transcription factors, including *atoh7*, *neurod4*, *crx*, *rorb*, and *foxn1*, were also shown to be simultaneously significantly downregulated. This finding may be related to the somewhat delayed growth of the mutant eyes (Fig. 8B). Then, GO term and KEGG pathway enrichment analyses were performed to retrieve gene functional profiles of upregulated or downregulated gene sets. The molecular functions (MFs), biological processes (BPs) and cellular components (CCs) were predicted for the DEG's significantly enriched GO terms. The GO terms indicated that the DEGs were involved in various processes, such as DNA integration, DNA metabolic process, response to stress, DNA recombination, intermediate filament cytoskeleton, polymeric cytoskeletal fiber, supramolecular complex, and supramolecular fiber (Fig. 8C). The KEGG pathway analysis of DEGs in *flrt2*-KO zebrafish eyes compared with WT found that they were enriched in the following pathways: Pantothenate and CoA biosynthesis, Pyrimidine metabolism, Fanconi anemia pathway, Drug metabolism-other enzymes, 2-Oxocarboxylic acid metabolism, linoleic acid metabolism and necroptosis. The enrichment of these pathways may be a result of *flrt2*-KO and WT eyes having slightly differing metabolic states (Fig. 8D).

## DISCUSSION

There have been prior reports that the *flrt* gene family is highly expressed in the developing embryo (Lacy et al., 1999). FLRT proteins possess an extracellular leucine-rich repeat motif that is essential for cellular behaviors, such as protein–protein interactions, cellular adhesion or repulsion, migration and growth (Kobe and Kajava, 2001), and they interact with a number of other proteins, including ROBO1 (Akita et al., 2016), LPHN3 and UNC5 (Lu et al., 2015; Seiradake et al., 2014). FLRTs perform their roles as virulence factors (Evdokimov et al., 2001), ribonuclease inhibitors, or splicing mediators (Price et al., 1998) through these interactions. As a member of the FLRT family of proteins, FLRT2 was initially discovered as a chemorepellent in neurons, and it functions as a repulsive ligand of the UNC5 receptor family. FLRT2 localizes in the mammalian brain throughout embryological development and has been linked to neural circuit creation, synapse formation, and neuronal migration (O'Sullivan et al., 2012; Yamagishi et al., 2011). Moreover, in E10.5 mouse embryos, FLRT2 is expressed in the inner (neural) layer of the OC, which corresponds to the future neural retina (Gong et al., 2009). This finding is similar to our results using microarrays to analyze the specific transcriptome of embryonic mouse retinal precursor cells, namely that *Flrt2* is highly specifically expressed during early retinal development (Cao et al., 2018b). In the current study, *flrt2* expression was also found by WISH in zebrafish embryos from 24 to 72 hpf, and its presence was shown in dorsal and lateral views, which further supports this conclusion.

When *flrt2* was knocked out by CRISPR/Cas9, the eyes and body of *flrt2*^−/−^ zebrafish larvae became much smaller and shorter than those of WT at 36 hpf, which implied that the absence of *flrt2* resulted in delayed and faulty larval development. The development of the embryonic eye includes optic vesicle formation, OC invagination, embryonic fissure closure, anterior and posterior chamber formation, and visual function maturation. If the OF cannot be formed, that is, an obstacle occurs during the invagination of the OC, then the clinical manifestation is microphthalmia or anophthalmia. However, the immunofluorescence staining of the retinal basement membrane showed that the process of the embryonic eye development in *flrt2*^−/−^ zebrafish larvae was normal. The retina's two sides moved in close proximity and pressed together at 36 hpf, and they were completely fused at 72 hpf. A reduction in organ size can occur due to an increase in early developmental cell mortality or a decline in progenitor cell proliferation. The BrdU labeling experiments demonstrated that the cell proliferation activity of the *flrt2*^−/−^ larvae was comparable to that of the WT at 36 hpf, which argued against abnormalities in progenitor cell proliferation as the source of the microphthalmia phenotype. The TUNEL assay revealed that *flrt2* deficient retinas had much higher levels of apoptotic activity. Accordingly, we inferred that cell death was one of the causes of microphthalmia. However, it is interesting to note that as the larvae mature, the faulty and delayed development in *flrt2*^−/−^ larvae becomes less obvious. Although the precise cause of this change is not yet known, it indicates that other compensatory systems may exist. In addition, the only impact of *flrt2* on eye development could be its effect on eye size. Because none of the key retinal cell types, including GCs, BCs, ACs, PRs, and MGCs, were impacted in *flrt2*^−/−^ zebrafish, there were no apparent phenotypes with altered retinal cell differentiation or development. The retinotectal pathway is involved in the processes underlying developmental emmetropization, and factors that affect emmetropization, such as myopia, may change eye size (Arumugam et al., 2016) or refraction (Tkatchenko et al., 2018). Unfortunately, in our study, we did not demonstrate an effect of *flrt2* on optic nerve pathway development. No changes in the axons of the optic nerve and the central longitudinal tracts were found in *flrt2*^−/−^ mutants and no axonal decussation abnormalities were exhibited. However, complicated mechanisms that govern axonal outgrowth and pathfinding in zebrafish are what govern the highly stereotyped retinotectal route. The supply of mitochondria, proteins, and other chemicals to nerve terminals via active intracellular transport is crucial for axonal expansion. Therefore, even if *flrt2* is involved in axon formation, cell migration, and adhesion, such as guiding cell motions or neurite outgrowth, we hypothesize that it may not be a primary regulator of optic nerve development.

Our RNA-seq data indicated that the apoptosis-associated genes, *Casp3a* and *Caspbl*, which from the apoptosis cascade pathway were upregulated. Consequently, we postulated that the activation of the apoptotic cascade pathway may cause cell death, which in turn may lead to the emergence of microphthalmia. In addition, this phenotype may potentially be influenced by the downregulation of certain genes involved in retinal development. *Rorb* is expressed in parts of the central nervous system that are thought to be crucial to their function, including the suprachiasmatic nuclei, the retina, and the pineal gland (Flores et al., 2007). A bHLH transcription factor called *Atoh7* is necessary for deciding the fate of retinal ganglion cells. The bHLH transcription factor *neurod*, which encourages the cell cycle exit of rod and cone progenitors, is also essential for the development of photoreceptors (Ochocinska and Hitchcock, 2009). The *otx* family member *Crx*, which is highly conserved, can aid in the differentiation and specification of retinal progenitors, including, but not limited to, photoreceptors (Shen and Raymond, 2004). Additionally, *pcdh8* plays critical roles in the formation of neurons and has a high capacity for intracellular signaling (Etzrodt et al., 2009). Microphthalmia may result from a lack of these genes. Therefore, we hypothesized that the combined effect of downregulating *crx*, *neurod4*, *atoh7*, and *pcdh8* and upregulating *Casp3a* and *Caspbl* may be responsible for the smaller eye size in the *flrt2*-KO zebrafish. Further research is necessary to determine the mechanism, but *flrt2* is likely the indirect regulator in this case. Moreover, it is uncertain how *flrt2* controls the expression of its downstream target genes and the development of the zebrafish retina. Ablation of the downstream target genes of *flrt2* will be the subject of further research as this will help to directly show the impacts of these genes.

Taken together, our study's findings suggested that *flrt2* is necessary for the normal development of the zebrafish eyes because they showed that it is strongly expressed in the retina and that the deletion of *flrt2* causes microphthalmia in zebrafish. Furthermore, the overexpression and downregulation of genes related to neural retinal development may be responsible for the defective and delayed development caused by the *flrt2*-KO and additional research is expected to corroborate the results of this study. Our results contribute to the knowledge of the function of *flrt2* in zebrafish retinal development and may encourage additional research in the areas of retinal development and evolution.

## MATERIALS AND METHODS

### Zebrafish husbandry and embryo preparation

Adult zebrafish was kept and bred in a separate system at Zhongshan School of Medicine, Sun Yat-sen University, Guangzhou, at a controlled temperature (28.5°C) and on a live and pellet diets. Embryos were collected through natural spawning, staged in hours post fertilization (hpf), days post fertilization (dpf), or months post fertilization (mpf). All of the manipulations were approved by the local ethical review committee at Zhongshan School of Medicine, Sun Yat-sen University.

### Generation of zebrafish *flrt2* mutants using CRISPR/Cas9

The *flrt2* gene (ENSDARG00000079355) was targeted with sgRNAs in accordance with the CRISPR/Cas9 principle using a CRISPR design tool (http://crispr.mit.edu/). In this study, the *flrt2* effective target sequence was GTAGAAACGGTTTACCTGTAC. At the one-cell stage, zebrafish embryos were injected with the injection mix, which contained 80 ng/µl gRNA and 500 ng/µl zCas9 mRNA. At 24 hpf, the embryos were screened to determine a founder, and the germline mutation frequency was shown to be approximately 20%. Mutant sites were confirmed using PCR and sequencing (comparison to WT unaffected sequences). The primers used for this part study are listed in Table 1. The PCR conditions were as follows: an initial denaturation step at 95°C for 5 mins, followed by 30 cycles of 95°C for 30 s, 58°C for 30 s, and 72°C for 1 min, followed by 10 min incubation at 72°C. The chimeric zebrafish were bred in the AB background to get *flrt2*^+/−^ zebrafish. Then *flrt2*^+/−^ male zebrafish and *flrt2*^+/−^female zebrafish were bred to get *flrt2*^+/+^, *flrt2*^+/−^and *flrt2*^−/−^ zebrafish, which were used in all the phenotypic analyses.

**Table 1.**
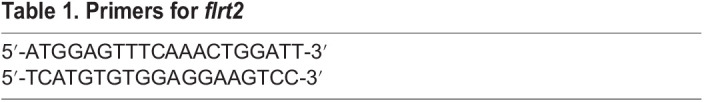
Primers for *flrt2*

### Quantitative real-time PCR (qRT-PCR)

Following the manufacturer's directions, total RNA from 30 embryos was collected using TRIzol Reagent (ThermoFisher Scientific), and from these RNA samples (approximately 1 µg in 20 µl), cDNA was synthesized. qPCR analyses were carried out in total reaction volumes of 5 µl containing 0.5 µl template cDNA, 2.5 µl SYBR Green I PCR Master Mix (Roche), and 5 µM of each primer. The reactions were performed as follows: pre-incubation step at 95°C for 5 min, followed by 45 cycles of amplification at 95 for 10 s, 60 for 10 s, and 72 for 10 s using LightCycler480 system (Roche). The control gene for normalization was *β-actin*. The primers used in this study are listed in Table 2. The relative expression of mRNA was calculated by the 2^−△△Ct^ method. The negative controls consisted of all the reaction components without template cDNA. For *flrt2*, the above experiment was performed in three independent duplicates.

**Table 2.**
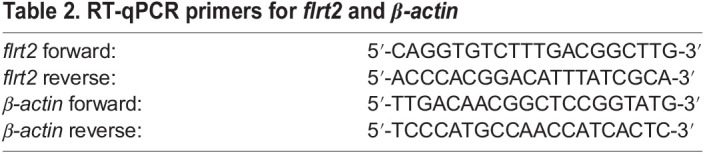
RT-qPCR primers for *flrt2* and *β-actin*

### In situ hybridization (ISH)

Zebrafish embryos were stored in 100% methanol at −20°C after being fixed in 4% paraformaldehyde (PFA) overnight at 4°C. WISH was conducted as previously described as follows: dehydrated embryos that have been gradually rehydrated using methanol dilutions in PBST (PBS with 0.1% Tween-20), followed by four rounds of PBST washing. Then, 10 µg/ml Proteinase K was used to digest embryos for 5 to 30 min at the room temperature. After stopping the digestive process, the embryos were incubated for 20 min at room temperature with 4% PFA before being washed four times with PBST to eliminate any remaining PFA. The embryos were transferred in sterile EP tubes measuring 1.5 ml, and then, they were prehybridized with 200 µl prehybridization solution for 1 h at 65°C. The prehybridization solution was discarded, and 100 µl of hybridization solution containing digoxigenin-labeled RNA probes was added into each tube. The tubes were then hybridized overnight at 65°C. The probes were recycled the following day, and the hybridization solution was gradually changed to 2× saline sodium citrate (SSC) through a series of 10-min washes at 65°C. Then, the tubes were washed twice in 0.2× SSC at 65°C. In the following step, all the tubes were washed at room temperature, and 0.2× SSC was progressively replaced with PBST. The embryos were first placed in the blocking buffer for 2–4 h before 200 µl of anti-digoxigenin antibody solution (Roche, 11093274910) that had been diluted 1:2000 with the blocking buffer was added, and the embryos were incubated overnight at 4°C. The antibody solution was discarded, and then, the embryos were washed six times in PBST at room temperature with gentle shaking. The embryos were transferred from the tubes to a 12-well plate after being gently shaken three times in an alkaline phosphatase (AP) buffer at room temperature. The AP buffer was removed, and 0.5 ml of freshly produced, dark-stored staining solution (BM Purple AP Substrate, Roche, 33300000) was added. A Leica stereomicroscope M205 was used to capture the images.

### Immunohistochemistry

The procedures were performed according to the previous report (Cao et al., 2018a). Briefly, zebrafish embryos were fixed in 4% PFA at 4°C overnight. They were then dehydrated in 30% sucrose until they sank, embedded in OCT tissue freezing medium, and cryosectioned at 10-μm thickness using a Leica Cryostat Microtome. Slides were subjected to antigen retrieval for 30 min at 98°C, except for those analyzed with anti-Zn5 antibodies. They were then incubated with primary antibodies overnight at 4°C, including anti-Zn5 (1:500, Zebrafish International Resource Center), anti-Pax6 (1:500, Covance, PRB-278P-100), anti-αPKC (1:500, Santa Cruz Biotechnology, sc-208), anti- GS (1:500, BD Biosciences, 610518), anti-GFAP (1:500, ThermoFisher Scientific, PA5-16291), anti-Recoverin (1:500, Millipore, AB5585), and anti-Rho 1D4 (1:500, Abcam, ab5417). Then, the slides were washed with PBST three times and incubated with Alexa Fluor 488-conjugated donkey anti-rabbit antibody (1:500, Invitrogen, A-10042) and Alexa Fluor 568-conjugated donkey anti-mouse antibody (1:500, Invitrogen, A-21202) at room temperature for 2 h. Images were obtained using a confocal microscope (Ism 710; Zeiss).

### Bromodeoxyuridine (BrdU) labeling and immunostaining

Using a glass micropipette, the 34 hpf zebrafish embryos were dechorionated and injected with approximately 0.5 nl of 10 mM BrdU (Sigma-Aldrich) to the heart in the yolk sac caudal. Embryos were injected, placed back in fish water for 2 h, harvested, and then subjected to the aforementioned immunostaining procedures using an anti-BrdU antibody (1:100, RPN20; GE Healthcare BioSciences, Pittsburgh, PA, USA).

### TUNEL assay

The TUNEL assay was carried out on cryosections of zebrafish embryonic heads using the In Situ Cell Death Detection Kit, Fluorescein (Roche, 11684795910), as directed by the manufacturer. Images were captured using an LSM 980 confocal microscope (Zeiss, Germany).

### Lipophilic dye labeling

Embryos treated with 1× phenylthiourea (PTU; 0.05 mmol/l; Sigma-Aldrich) were fixed in 4% PFA at 6 dpf. Following agarose immobilization on glass slides, they were injected with DiI (1 mmol/ml, Invitrogen, V22885) and DiO (1 mmol/ml, Invitrogen, V22886) into the vitreous chamber of the retina. After the dye had spread to the tectum, the embryos were subsequently incubated for 24 h. Using an LSM 980 confocal microscope, the Z-axis was used to record RGC axon projection.

### RNA sequencing and analysis

The eyes from 36 hpf zebrafish embryos were collected and placed into TRIZOL Reagent (ThermoFisher Scientific). Approximately 40 eyes were pooled together as one sample. The eight samples, four WT and four *flrt2*-KO zebrafish were sequenced. Following the manufacturer's instructions, sequencing libraries were created using the NEBNext Ultra RNA Library Prep Kit for Illumina (NEB, MA, USA). The libraries were sequenced on an Illumina HiSeq platform, and 150 bp paired-end reads were generated. Raw data were filtered to remove low-quality reads, and the clean reads were aligned to the zebrafish reference genome (GRCz11) using Hisat2 v2.0.5 (Kim et al., 2015). Then, featureCounts v1.5.0-­p3 (Liao et al., 2014) was used to count the number of reads mapped to each gene, and the fragments per kilobase of transcript sequence per millions base pairs (FPKM) of each gene were calculated. The DESeq2 R software was used to perform differential gene expression (Love et al., 2014). DEGs were defined as genes with an adjusted *P*-adj 0.05 and a fold change >1. GO term and KEGG analyses were carried out with the aid of the DAVID bioinformatics tools (Huang et al., 2009).

### Statistical analyses

The results of at least three independent experiments are presented as the means±standard deviations and were compared using two-tailed Student's *t*-tests. The results were analyzed by GraphPad Prism (version 9, San Diego, CA, USA).
